# Early identification of young children at risk for poor academic achievement: preliminary development of a parent-report prediction tool

**DOI:** 10.1186/1472-6963-11-197

**Published:** 2011-08-18

**Authors:** Susmita Pati, Kyleen Hashim, Brett Brown, Alexander G Fiks, Christopher B Forrest

**Affiliations:** 1 Division of Primary Care Pediatrics, State University of New York at Stony Brook and Stony Brook Long Island Children's Hospital, Stony Brook, NY, USA; 2 Leonard Davis Institute of Health Economics, University of Pennsylvania, Philadelphia, PA, USA; 3 Presidential Management Fellow, Department of Housing and Urban Development, Washington DC, USA; 4 Walter R. McDonald & Associates, Inc., Rockville, MD, USA; 5 Center for Clinical Epidemiology and Biostatistics, University of Pennsylvania, Philadelphia, PA, USA; 6 Division of General Pediatrics, The Children's Hospital of Philadelphia, Philadelphia, PA, USA

## Abstract

**Background:**

Early school success is clearly related to later health. A prediction index that uses parent report to assess children's risk for poor academic achievement could potentially direct targeted service delivery to improve child outcomes.

**Methods:**

We obtained risk factors through literature review and used the National Longitudinal Survey of Youth 1979 Child Files to examine the predictive associations of these factors with academic achievement scores.

**Results:**

Twenty predictors were identified including four strong predictors (maternal education, child gender, family income, and low birth weight). Significantly, 12 predictors explained 17-24% of score variance.

**Conclusions:**

Parent-reported factors provide predictive accuracy for academic achievement.

## Background

Early school success is clearly related to later success and health [1,2]. Promoting optimal child health and development increases the likelihood of school success and is therefore important not only for children's immediate outcomes, but also for their future. Over the past 20 years, strategies to strengthen child health supervision in pediatric settings through programs such as *Bright Futures *[3] and *Healthy Steps *[4] have been developed. Although *Bright Futures *recommends that "more frequent visits may be indicated for children at increased risk because of medical and/or social concerns," [3] health professionals do not have standardized, evidence-based approaches to identify at-risk children and families. As a consequence, the current provision of health supervision is formulaic despite differences in children's underlying needs. Identifying children at different levels of risk is the first step in tailoring preventive care to promote early school success and, in turn, later health.

New methods are required to better identify and match a child's need for preventive services with actual provision of those services, such that more intensive developmental and health assessments are done for those at greatest risk of poor future outcomes. Researchers have produced a rich scientific base on the risks that are predictive of early school success comprised of readiness to learn in school [2,5,6], successful coping in school settings [7], and child health and well-being [8]. However, very little information exists on the magnitude of effects of various risk factors and their cumulative contribution in predicting early school success.

The purpose of this manuscript is to determine the feasibility of constructing an early school failure risk index using data that can be obtained solely from parents within a primary care setting. The development of this tool is the first step in a research program ultimately designed to develop a practice-based prediction tool for early school success in order to assist clinicians in tailoring pediatric health supervision visits or other services coordinated through the medical home to individual child needs. A prediction tool that assesses a young child's risk profile could inform and support clinician's decisions regarding preventive care, such as scheduling more frequent (or longer) office visits, making early intervention referrals, or involving other community supports for at-risk children identified by the tool. To enable widespread use, such a prediction tool should be easily administered and scored in busy office settings. This suggests the need for a limited set of risk factors that can be easily obtained from parental report. In this first step of the research program, we obtained an initial set of evidence-based risk factors through literature review and used the National Longitudinal Survey of Youth 1979 Child Files to examine the predictive associations of the risk factors, assessed at age two, with school success assessed by achievement scores at age six or seven.

## Methods

### Conceptual Framework

**Figure 1:**
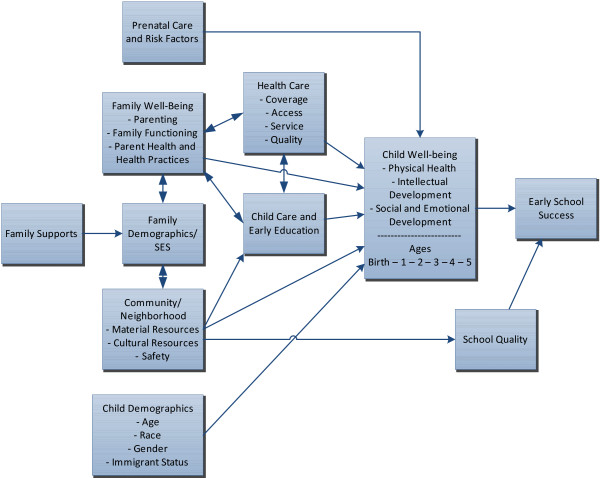
**Model of Early Childhood Development Leading to Early School Success**. Source: Child Trends: Based on a model presented in Brown, B., Weitzman, M., Bzostek, S., Kavanaugh, M., Aufseeser, D., Bagley, S., Berry, D., Auinger, P. (2004). *Early Child Development in Social Context: A Chartbook*. New York: The Commonwealth Fund.

The framework guiding our selection of predictors to create this index was based on a model originally adopted by the National Education Goals Panel, and subsequently refined in the work of Zaslow et al. [6] and Brown et al. [9] (Figure 1). The framework is developmentally appropriate, focuses on the whole child, and incorporates an ecological perspective that recognizes the ways in which family and community can influence early development. The model has been widely adopted in early development and school readiness research [2,5,6].

### Initial Item Pool Creation

Guided by our conceptual framework, we identified an initial set of 68 predictors of early school success based on a structured review of the literature as referenced in Table 1[2,5,10-37]. Details of the methodology for the literature review are presented in Appendix A. Because our goal was to obtain a parsimonious set of predictors, we categorized the evidence for each predictor as strong, moderate, weak, or mixed based on the strength of association in large population studies [38].

**Table 1:** Predictors of Early School Success Identified from Literature Review*

Relative size of effect	Predictor
**Strong**	○ Maternal Education [12,16,18,19,32,56]
	○ Gender [12,15,20,23,24,31,32]
	○ Family income [15,16,20,23,27,29,32,36,56]
	○ Low birth weight [10,12,13,19,20,23,25,29,31,32]

**Moderate**	○ Prematurity [23,31]
	○ Prenatal cigarette exposure [13,15,20]
	○ Maternal affect [27,31]

**Weak**	○ Maternal age [12,15,19,20,23]
	○ Parental cognitive ability [15,20]
	○ Maternal warmth [30]
	○ Maternal sensitivity [56]
	○ Punitive parenting [30]
	○ Television viewing [37]
	○ Prenatal care [15,19]
	○ Early hospitalization [29]
	○ Second-hand smoke [20,31]

**Mixed**	○ Race/ethnicity [12,15,20,24,29,32] (mostly strong, but weak or not significant in multivariate adjustment)
	○ Family structure [12,23,24,32](mixed, mostly strong)
	○ Family size [24,31,32] (two studies are moderate to strong, one study not accounting for family structure is mixed)
	○ Prenatal alcohol exposure [20] (mixed-weak to moderate for reading & NS for math)

**Other potentially important factors in the literature**	○ *Sociodemographics*: parental literacy, parental health literacy, birth intervals, immigrant status, English proficiency, parental employment
	○ *Prenatal/childhood medical problems*:
	■ *Health care*: age-appropriate pediatric care, age-appropriate immunizations
	■ *Nutritional deficits*: failure to thrive, underweight, iron deficiency
	■ *Early special medical care/chronic conditions: *visual ability, ear infections, low APGAR
	■ *Development*: early language and literacy skills, cognitive ability, developmental disability, motor skills, deafness, speech defects
	○ *Behavior and personality characteristics*: internalizing and externalizing behavior, social functioning, attention, self-regulation, affect, temperament
	○ *Social environment*
	■ *Prenatal environment*: maternal mental health, unintended pregnancy
	■ *Home environment*: lead exposure, family conflict, number of books, reading to children
	■ *Parenting*: attachment, developmental and educational expectations, exposure to speech
	■ *Child care*: type, provider ratio, provider education, classroom features, accreditation, hours
	○ *Neighborhood conditions*: poverty, affluence, male joblessness, safety

* Details regarding literature review, data extraction, and predictor classification are presented in Appendix A. In brief, predictors were categorized as strong, moderate, or weak based on the strength of the association (or effect size) with early school success in relation to the scale of the predictor. For example, a predictor with a broad range (e.g., income) was considered strong if the beta was 0.1 whereas for a predictor with a narrow range (e.g., gender), a beta greater than 2.5 was considered strong.

### Data Source

To select the data source, we examined several longitudinal data sets, including the Panel Study of Income Dynamics- Child Development Supplement, National Longitudinal Survey of Youth 1979 (NLSY79) Child Files, Early Childhood Longitudinal Study-Kindergarten Cohort, Early Childhood Longitudinal Study-Birth Cohort, and Head Start Family and Child Experiences Survey for three criteria: 1) appropriate age span (from age two through school age); 2) a large probability sample; and 3) a wide range of relevant predictor and outcome measures. The NLSY79 child file, a longitudinal dataset that is generally representative of all children born to young, baby boomer mothers, was selected because it best fit these criteria [39]. We used seven cohorts from the NLSY79 Child Files for this analysis. The analytic sample consisted of 2919 children who were age two in one of 7 rounds conducted between 1986 and 1998 (rounds were conducted every other year) and who were also followed up at six or seven between 1990 and 2004.

### Predictor Variables

We focused on predictors at age two because identification of factors in early childhood presents opportunities for tailoring pediatric health supervision and referring children to early intervention or other services to promote future school success. Moreover, contact between pediatricians and families is greatest in the first two years of life and, by age two, a child has accrued sufficient development history for parents to be able to report on a diverse set of milestones.

From our initial pool (n = 68), 31 items/multi-item subscales were available in the NLSY and all but one (the multi-item HOME-Short Form) relied solely on parental report (Table 2). The predictors available in the data-set included all those categorized as strong or mixed, all but one categorized as moderate, and most of those categorized as weak in Table 1. Of note, because no neighborhood or health care receipt predictors were significant in our final models, the remaining predictors were classified into 3 domains (child characteristics, family characteristics, and home environment) for ease of translation into practice; this domain classification is utilized in Tables 2 and 3. We combined 2 items, family structure and conflict about child rearing, into a single item because the item about conflicts was not asked of single-parent families. Two predictors were multi-item scales that have been previously validated (i.e. the Center for Epidemiologic Studies-Depression Scale [40,41] and the HOME emotional subscale [42]) and several were multi-item scales specifically developed for use in the NLSY based on existing scales (i.e. Motor-Social Development Scale, Brief Compliance Scale, Brief Indicators of Insecure Attachment). In addition to the composite scores, results are presented for selected items within the HOME cognitive stimulation subscale (i.e. "number of books" and "frequency of reading to child"), Motor-Social Development, and Brief Compliance Scale documented in the literature as potentially important stand-alone items that were used in subsequent analytic model development.

**Table 2:** Characteristics of two-year old children sampled in the National Longitudinal Survey of Youth 1986-1998 (n = 2919 unweighted)

CHILD CHARACTERISTICS	
***Child demographics***	

**Female**	49.2%
**Number of children in household**	
1 (focal child only)	25.9%
2-3 children	63.3%
4 or more children	10.8%
**Birth order (mean)**	2.1

***Physical health factors***	

**Gestational age**	
Very premature (< 32 weeks)	1.3%
Premature (< 37 weeks)	12.3%
**Birthweight**	
Very low birthweight (< 1500 grams)	0.6%
Low birthweight (between 1500 grams and 2500 grams)	6.5%
**Weight at age two**	
Body Mass Index < 5^th ^percentile	17.2%
Body Mass Index > 95^th ^percentile	14.3%

***Developmental abilities***	

Motor-Social Development score^1 ^(mean)	103.3
Child has named 4 colors	64.4%
Child has counted from 1-10	47.3%
Has gone to the toilet alone	72.0%

***Personality***	

Brief Compliance Scale score^2 ^(mean)	22.1
Almost always obeys when told to turn off TV	60.7%
Never resists going to bed	30.5%
Brief Indicators of Insecure Attachment score^3 ^(mean)	19.8

**FAMILY CHARACTERISTICS**	

**Mean maternal age at child's birth**	27.2 years
**Maternal age at child's birth**	
< 25 years	25.8%
25-29 years	43.9%
≥ 30 years	30.3%
**Maternal race/ethnicity**	
Non-hispanic white	63.0%
African-American	20.3%
Hispanic	13.3%
Asian/Pacific Islander	0.6%
Native American	1.7%
Other	1.2%
**Mother U.S. Born**	95.1%
**Maternal interview conducted in English**	98.8%
**Family structure at age two**	
Married	75.1%
Cohabiting with partner	5.5%
Other	19.5%
**Maternal employment status^4^**	
Full-time	35.5%
Part-time	16.5%
Not working	48.1%
**Living at or above poverty^5^**	79.9%
**Maternal Educational Attainment**	
Less than High School	17.7%
High School	40.2%
Some college	21.4%
Bachelor's degree or beyond	20.8%
**Maternal depressive symptoms (CES-D score ≥ 16)^6^**	22.3%
**Intendedness of pregnancy^7^**	
Wanted pregnancy	67.7%
Mistimed pregnancy	23.9%
Unwanted pregnancy	8.4%

**HOME and NEIGHBORHOOD ENVIRONMENT**	

***Prenatal environment***	

No prenatal cigarette exposure	69%
Prenatal alcohol exposure	
No alcohol	64.7%
Moderate alcohol (< 1/month to < 3-4 days/month)	30.6%
Heavy alcohol (1-2 days per week or more)	4.7%

***Child care type in third year of life***	

Parent	52.4%
Relative	14.1%
Nonrelative	14.0%
Center-based care	19.3%
Other	0.2%

***Parenting environment***	

**Mother smokes daily**	29.5%
**Number of books child has of his/her own**	
< 3	9.3%
3-9	17.0%
≥ 10	73.7%
**Frequency of reading to child**	
Less than three times per week	29.4%
Three times per week	31.6%
Daily	39.0%
**Hours watched TV on average school day (mean)**	3.0 hours
**How often does mother argue with spouse/partner about child rearing?**	
No spouse/partner	16.6%
Never/hardly ever &**CHILD CARE**	46.8%
Often/sometimes	36.6%

***HOME Emotional Subscale^8^***	

Mother talks to child while working	
Always	44.0%
Often	44.2%
Sometimes	10.5%
Rarely	1.1%
Never	0.3%
Number of times mother spanked child during the past week (mean)	2.3
Mother kissed/hugged child (interviewer observation)	75.9%
Mother spoke spontaneously to child (interviewer observation)	93.3%
Mother verbally responded to child (interviewer observation)	92.4%
Mother restricted child 4 or more times (interviewer observation)	19.9%

***Neighborhood characteristics***	

**Social connectedness: People keep to themselves and don't care what goes** **on in the neighborhood**	
Big problem	6.3%
Somewhat of a problem	23.3%
Not a problem	70.3%
**Joblessness: Lots of people can't find jobs**	
Big problem	13.3%
Somewhat of a problem	16.7%
Not a problem	70.2%
**Crime: Is crime and violence a...**	
Big problem	6.2%
Somewhat of a problem	20.2%
Not a problem	73.7%

^1^Created by the National Center for Health Statistics, the Motor-Social Development Score (MSD) measures aspects of young children's motor, social, and cognitive development and was derived from items in the Bayley Scales of Infant Development, Gesell Scale, Denver Developmental Screening Test, and other child developmental scales. The measure consists of 15 age-appropriate, maternal response items. Scores were standardized (mean = 100, std dev = 15) and normed by age and gender.

^2^Brief Compliance Scale. This 6-item scale created for the NLSY is based on maternal reports of a child's typical behavior. Items include whether the child resists/obeys expectations for eating, going to bed, or watching television. Raw scores ranged from 5-34.

^3^Brief Indicators of Insecure Attachment. This 7-item subscale created for the NLSY is based on maternal reports of a child's typical behavior. Items include whether the child becomes upset when the mother leaves, is difficult to soothe, stays close while playing, is empathetic/demanding, copies other's actions, and wants to help. Raw scores ranged from 5-39.

^4^Mothers were asked if they worked at all during the past week. Those who reported they could not work or did not work were classified as "not working." Among those working, mothers were asked if they usually worked "full-time" (> 35 hours per week) or "part-time" (< 35 hours per week).

^5^Poverty status is based on annual Poverty Income Guidelines issued by the Department of Health and Human Services. This poverty definition is a similar but simplified version of the Federal Poverty Level where family size in general is considered but the number of children or elderly is not specifically taken into account. http://aspe.hhs.gov/POVERTY/faq.shtml#official

^6^The Center for Epidemiologic Studies Depression scale (CES-D) was administered to mothers at the time of the interview at age two. Mothers with a score of 16 or higher were classified as having depressive symptoms in accordance with the DSM-IV criteria for clinical depression.

^7^Mothers were asked if they had wanted to become pregnant before their pregnancy. "Yes" or "didn't matter" was coded as wanted, "not at the time" was coded as mistimed, and "not at all" as unwanted.

^8^The HOME Emotional subscale represents a subscale of a modified HOME inventory that measures the emotional support provided by the child's mother. Scores are standardized (mean = 100, standard dev. = 15) and based on a combination of maternal responses and interviewer observations. Items from the HOME emotional subscale were individually selected for use in subsequent multivariate regression models as approximations of maternal warmth, maternal responsiveness/sensitivity, and harsh discipline because the broader literature suggests these factors are predictive of early school readiness.

**Table 3:** Multivariable regression results for predictors available at age two associated with PIAT scores in math, reading recognition, and reading comprehension at age six or seven

Predictor	School	Success	Outcomes
	**Math** **(n = 2200)**	**Reading Recognition** **(n = 2178)**	**Reading Comprehension** **(n = 1384)**
**Adjusted R^2 ^**	0.24	0.19	0.17
**Intercepts**	87.68	87.18	96.24

**CHILD CHARACTERISTICS**			

***Child Demographics***			

**Female**	0.30	**2.04**	**1.50**
**Number of children**			
1 child		Reference	
2-3 children	-1.21*	**-2.11**	**-1.55**
4 or more children	**-2.85**	**-3.96**	-2.08*

***Developmental abilities***			

**Motor-Social Development score**	**0.12**	**0.09**	**0.06**

***Personality***			

**Brief Compliance Scale score**	**0.19**	0.11*	0.03

**FAMILY CHARACTERISTICS**			

**Maternal age at child's birth**	0.09	**0.30**	**0.30**
**Maternal race/ethnicity**			
Non-hispanic white		Reference	
African-American	**-4.57**	-0.41	-0.92
Hispanic	**-2.28**	0.38	-0.39
Asian/Pacific Islander	**1.13**	7.57	4.13
Native American	-9.26	-4.96*	4.21
Other	-0.13	**2.04**	1.50
**Maternal interview not in English**	-4.53	-5.57	0.24
**Maternal educational attainment**			
Less than high school	**-9.16**	**-7.11**	**-5.22**
High school	**-5.86**	**-3.80**	**-3.26**
Some college	**-2.95**	-1.53*	-0.90
Bachelor's degree or beyond		Reference	
**Income (in dollars)**	5.52*E^-7^	1.08*10^-5^	4.70*10^-6^

**HOME ENVIRONMENT**			

***Family structure & conflict over child rearing***			

Married/Partner Low Conflict		Reference	
Married/Partner High Conflict	**-2.06**	-1.20	**-2.12**
Married/Partner Unknown Conflict	-0.99	**-1.69**	**-3.66**
Unmarried/No partner	**-3.22**	**-2.11**	**-2.70**

***Number of books child has of his/her own ***			

< 3		Reference	
3-9	**2.30**	1.72*	0.59
≥ 10	**2.65**	2.06*	0.25

***Frequency of reading to child***			

Less than three times per week		Reference	
Three times per week	0.44	0.06	1.42*
Daily	**1.67**	0.47	1.56*

**Notes: **Beta values from weighted regression models are presented for each predictor. In the entire NLSY79 Child Files encompassing the rounds from which we drew our sample, approximate response rates for Math and Reading Recognition scores were 92 percent and 91 percent, respectively [47]. Reading Comprehension responses rates were typically lower, ranging from 86 percent to 91 percent [47]. This resulted in sample sizes for our analyses of six and seven-year olds of 2200 for math, 2178 for reading recognition, and 1384 for reading comprehension. All values in bold are significant at p < 0.01.

* p < 0.05.

### Outcome Variables

We operationalized school success as academic achievement measured with Peabody Individual Achievement Test (PIAT) scores at ages six and seven in Math, Reading Recognition, and Reading Comprehension. The PIAT is a standardized measure of academic achievement for children over age five [43-46]. It assesses reading and math skills within the child's range of difficulty. Though its primary purpose is to evaluate students referred for special education, it is among the most widely used brief measures of academic achievement in the literature and requires only a pointing response from children for most items [43-46]. Standardized scores have a mean of 100 with a standard deviation of 15. These scores have been found to be reliable and valid measures of academic achievement [43-46]. In the entire NLSY79 Child Files encompassing the rounds from which we drew our sample, approximate response rates for Math and Reading Recognition scores were 92 percent and 91 percent, respectively [47]. Reading Comprehension responses rates were typically lower, ranging from 86 percent to 91 percent [47]. This resulted in sample sizes for our analyses of six and seven-year olds of 2200 for math, 2178 for reading recognition, and 1384 for reading comprehension.

### Statistical Analyses

#### Analytical Model Development

The goal of the statistical analyses was to obtain the most parsimonious predictive model for each PIAT score in order to identify predictors to include in the summary risk index. To achieve this goal, we performed a series of statistical tests to narrow the number of predictors in each model (Appendix B). We began with bivariate associations between each of the 31 predictors and three PIAT outcomes, then examined multivariate regression models using child, family, or home variables only (included as categorical or continuous variables), and ultimately produced three separate predictive multivariate models, one for each PIAT score. At each step, we eliminated predictors that did not demonstrate a significant relationship (p < 0.05) with at least one of the three PIAT scores. All multivariate models were weighted per NLSY technical guidance [48]. Missing observations from the predictors in our multivariable regression models were handled according to the proportion of missing data as detailed in Appendix B [49,50].

## Results

Our sample includes 2919 two-year old children sampled in the NLSY between 1986 and 1998. At age two, most of these children lived in households with married parents and at least one sibling (Table 2). Child characteristics related to physical health and developmental abilities showed distributions for birth weight and prematurity consistent with nationally representative studies.

The family characteristics for sampled children showed a mixed picture of positive and negative factors. The majority of mothers were less than 30 years old, non-Hispanic whites, born in the United States, and spoke English as their primary language. Though more than 40% of mothers had completed some college, nearly half of them were not working when the child was two years old and just fewer than 21% of families were living in poverty. Nearly 70% of mothers had planned this particular pregnancy and more than 80% had prenatal care in the first trimester. More than 20% of mothers screened positively for depressive symptoms and approximately 37% reported that they lived in high conflict households where arguments with partners about child rearing occurred "sometimes" or "often."

Table 3 presents the results from our final multivariate regression models. These predictive models explained 17-24% of the observed variance in PIAT scores using 12 characteristics easily obtained from parental report. In these final models, nearly all child and family sociodemographic characteristics remained strongly associated with PIAT scores. However, among the other child factors examined, only the child's motor-social development and temperament scores related to compliance at age two remained predictive of PIAT scores whereas birth weight, overweight, and insecure attachment were not significant in the final models. Among factors examined in the home environment, reading to the child regularly and often as well as having a large number of children's books in the household remained highly predictive of PIAT scores. In contrast, the HOME emotional subscale, maternal smoking, and type of child care were not significant predictors of PIAT scores. Of note, maternal education and motor-social development were the only variables shown to be significant predictors for all outcomes.

## Discussion

Using data from the National Longitudinal Survey of Youth Child Files, we found that parental report on 12 characteristics of two-year old children can be compiled into a summary index that is predictive of early academic achievement. With further validation, this index could be used in clinical practices as a tool to assess risk for early school failure among preschool children and to assist clinicians' in determining the scope and depth of needed preventive care services. This is the first step in a research program whose overarching goal is to assist clinicians in tailoring pediatric preventive care to individual child needs.

The next step in this research program is to test the validity, feasibility and utility of this risk index in a pediatric ambulatory care setting. The instrument's predictive ability is only useful if it can feasibly be administered both effectively and efficiently. To date, we have successfully field-tested an expanded and modified version of this instrument in a cohort of 2100 families attending busy, urban pediatric practices and have found these items can be easily completed by parents in less than 10 minutes. Moreover, we are specifically examining whether information obtained from administrative and/or electronic medical records can be complemented by an even more parsimonious set of parental report items and still retain reasonably good predictive accuracy in sorting children into different groups of risk for early academic difficulties.

For practicing clinicians, early school success is currently often monitored during routine health supervision visits in an unstructured, informal manner. In 2006, the American Academy of Pediatrics issued an algorithm that recommends developmental surveillance at every preventive visit and prompt screening if problems are detected complemented by routine screening at the 9, 18, and 30 months visits [51]. With further validation and possible modification for different age groups, the prediction tool we have developed could provide a more efficient method of identifying children needing more frequent developmental screening. Notably, as with other clinical decision support tools (e.g., medication order entry, asthma management, etc.), clinicians may choose not to use results generated by the prediction tool; this is an important aspect to consider in assessing the potential impact, practicality, and optimal implementation strategy for the tool.

If the tool retains validity and feasibility in practice, the next step is to tailor preventive care to the needs of the child. For instance, for children in higher risk groups, clinicians could schedule more frequent or longer preventive care visits, make referrals to early intervention services or programs in the community, or complete more in-depth developmental assessments. Ideally, preventive care services offered to at-risk children should be evidence-based, high quality, and cost-effective. Though the current evidence base in this area is quite limited, the Agency for Healthcare Research and Quality has recently established 7 pediatric quality measurement centers of excellence that aim to advance this field [52]. We anticipate that the distribution of risk groups will vary from practice to practice and may change over time within any given practice. For those practices with greater proportions of at-risk children, providers may need to schedule longer and more frequent appointments, create effective collaborative relationships with local community-based early intervention agencies and other community-based support services, or hire staff (e.g., case managers, social workers, home visitation nurses, etc.) in order to address the needs of these children and families. Clinicians routinely judge patients' disease severity and other health risk factors to determine the mix and intensity of services that should be applied on an individual basis. For example, the NHLBI asthma severity groups (mild intermittent, mild persistent, etc.) sort patients from lowest to highest asthma severity level [53,54]. These strata are used to tailor pharmacological, disease monitoring, and specialty referral approaches for each patient. Disease and care management programs also use statistical models based on diagnoses and prior health service use to forecast which patients will have the greatest need and demand for services. These scores are used to stratify the intensity level of care management interventions; for example, the highest risk patients are contacted by nurse care managers multiple times each week, moderate risk patients less frequently (e.g., once a week), and lower risk patients receive standard educational services and periodic assessments.

Larger scale system changes may also be implemented to ensure effectiveness and sustainability of this model. For example, results of this tool may impact the partnership structure between the family and primary care medical home (e.g. the highest risk families have a nurse assigned to them; [55]), decision support (e.g. the highest risk families receive preferentially generated automated reminders), the patient/family education process (e.g. high risk families would receive specific education and training), and provider education (e.g. specific training for medical providers). Additionally, the current uniform payment scheme for pediatric preventive care would need to be adjusted to reflect the differences in provision of care among these groups.

In developing this prediction tool, we found that sociodemographics (i.e. maternal education, age, marital status, and child gender) and a few specific attributes of the home environment (i.e. number of children's books, frequency of reading to child, and parenting conflicts) were stronger predictors of early school success than child personality or developmental factors. Elucidating the mechanism by which these factors affect school success is one of the most persistent and challenging problems in this field. It is important to note that many of these factors have been repeatedly shown to have effects on child health and well-being that persist into adulthood [13,23]. Although this article does not assess the influence of other factors (e.g. preschool enrollment) that may be driving achievement scores, we intend to explore these relationships in the subsequent phases of our work.

Our primary goal was to develop a risk assessment tool that is predictive of early school success using parental-report. Aside from our primary goal of producing a summary instrument, we detected a few specific associations that merit additional comment. First, our finding that the home reading environment is an important predictor of early school success supports continued investments in initiatives to promote early literacy in the clinical setting. Reach Out and Read is one of the most cost-effective broad-based strategies adopted by clinicians to accomplish this goal. Pediatricians and child health professionals advise parents to read daily to their children and provide developmentally appropriate books at each of the 10 health supervision visits between ages 6 months to 5 years. In addition to early reading, a recent study utilizing six longitudinal datasets found school-entry math and attention skills were the strongest predictors of later academic success [56,57]. Taken in concert with our findings and others, clinicians should continue to encourage parents to enroll children in high quality preschool programs such as Head Start. Second, consistent with much work on racial/ethnic disparities in academic achievement [58], we found that race/ethnicity is an important predictor of early school success. However, the mechanisms underlying these persistent disparities are not well understood and further work in this area is required.

There are several caveats to this study. First, we define risk only in terms of factors predicting early academic achievement. If other outcomes were chosen such as health status or health risk, a different though overlapping predictor set would likely be identified. Such work would be a useful complement to this study. Second, these results are derived by studying two-year-old children. While this is an appropriate and early point for intervention and tailoring of services to preventive care needs, there is also an argument to be made for screening at an earlier age. Given that more than half (i.e. seven) of the important risk factors in our model could also be measured at birth, birth-screeners could also be developed with a modest amount of additional research. Developmentally specific summary indices would include additional age-appropriate items.

Although the NLSY-79 child file has one of the most complete sets of relevant risk measures available, we were not able to establish causality, model all of the risk factors identified in the research literature, or examine factors noted to be strengths. In particular, literature and developmental experts have noted that other parenting and home environment factors (e.g., parental health literacy, family social support, and parental aggravation) may be predictive of early cognitive outcomes [6]. Given that regular health supervision may improve early detection and treatment of problems related to school success, health care utilization patterns should also be considered for inclusion in any future summary risk index. In the subsequent phases of our work, we intend to explore the relationships between early school success and these omitted factors.

The risk index predicted between 17-24% of the variation in children's early academic achievement, and supports use of the tool for group-level analysis. Thus, the tool should be thought of as an instrument that produces scores that can be used to define groups of children by risk level. This level of explained variance is comparable to or higher than prediction models that are used for risk-adjusted provider payment and to identify future high-cost patients in need of care management [59]. We expect that an expanded set of predictors will explain a larger share of variation.

## Conclusions

To conclude, we created a prediction tool using 12 characteristics easily available from parental report at age two that could help customize the delivery of preventive care services in early childhood. By identifying at-risk children and families using a summary index (as compared to specific factors), the current one-size-fits-all approach to pediatric preventive care could eventually be transformed to provide different levels of services tailored to the specific preventive and developmental needs of children. Further work to integrate the fields of child development and health services research will advance our ability to identify children at-risk for early school failure efficiently and provide effective interventions. Developing appropriate tools to measure early school success in clinical practice is a critical step in this process and will ultimately improve our ability to ensure optimal child health and well-being.

## Competing interests

The authors declare that they have no competing interests.

## Authors' contributions

SP conceived of and designed the study, supervised the statistical analyses, and drafted the manuscript. KH participated in the design of the study and performed the statistical analyses. BB participated in the conception and design of the study and helped to perform and supervise the analyses. AF participated in the interpretation of the analyses and critically revised the manuscript for important intellectual content. CF participated in the conception and design of the study, supervision of the analyses, and helped draft the manuscript. All authors read and approved the final manuscript.

## Authors' Information

At the time of initial manuscript submission, Dr. Pati was a faculty member in the Division of General Pediatrics at The Children's Hospital of Philadelphia and the University of Pennsylvania's School of Medicine in Philadelphia, PA and Ms. Hashim and Dr. Brown were employed by Child Trends, Inc. in Washington, DC.

## Appendix A

### Two-Tiered Selection of Articles/Data Extraction

1^st ^Tier-Because there have been several excellent reviews cataloging correlates of school success, we began the literature review using fourteen major reviews that focused on specific domains of school readiness (e.g., emotional health, language, etc.) or risks/assets as the primary source documents. From these articles, we used a "snowball" approach whereby other references were obtained from reference lists of the fourteen articles and examined for relevance to this review. From each article identified, references were again reviewed for topic relevance. In addition, we used a major search engine to identify any other relevant articles using key words associated with general risks and outcomes, such as "risk", "resilience", "cognitive achievement", and "school readiness." All types of studies, including observational studies, were included in our review.

From the articles we identified in the 1^st ^Tier, we then selected articles based on the criteria detailed in Table 4. To ensure relevance to the primary question, we included only articles that measured one or more of the predictors of early school success among pre-school children. We also included only studies with large sample sizes primarily to identify factors with relatively high prevalence and to avoid problems of limited generalizability or unstable estimates. Finally, we limited our definition of school success to the cognitive dimension, including intellectual capacity or academic achievement.

**Table 4:** Inclusion criteria for articles

1. Sample size greater than 500, and *not *limited to a specific group by design (e.g., only children of a specific race, poverty level, or program)
2. Measured 1 or more of the predictors of early school success among children prior to school entry

3. Longitudinal studies with initial assessment prior to school entry and with follow-up at least 6 months later

4. Follow-up at age 54 months (4.5 years) or beyond

5. Assessment of cognitive capacity or academic achievement at a follow-up

6. Articles published during or after 1980.

A structured abstraction form was used to evaluate the articles. The form recorded citation, description and size of sample, predictors examined and measurement properties (i.e., validity and reliability, when available), covariates examined, and effect size (for those with the exposure and those without). Because of the complexities of comparing effect sizes across each review article, we created an additional table that ranks each predictor's strength based on its relative effect size, outcome, accompanying controls, and consistency in strength across studies.

Each predictor was assigned one of five possible rankings, including strong, moderate, weak, mixed, not significant, and no evidence. Owing to the small number of studies reporting odds ratios or risk ratios, the ranking process often involved relative comparisons of regression coefficients.

For example, Table 5 demonstrates the ranking process where the outcome scale (e.g., PIAT score) contains a range of 50 or more possible categories and the predictor scale ranges from 2 categories to 30 or more categories. For outcomes with a narrower range than the PIAT, smaller regression coefficients were given greater weight in determining relative ranking. We ranked predictors in this manner in order to identify factors with potential utility in developing interventions to improve early school success as well as utility in developing interventions to provide enhanced health care services to at-risk children and families.

**Table 5:** Ranking of Predictors

Rank	Predictor Scale (# of categories)	Outcome Scale (# of items in range)	Beta
**Strong**			
	2-3 (e.g., gender; LBW)	50+ (e.g., PIAT)	≥ 2.5-3.0
	8-15 (e.g., edu; income in 10,000's)	50+ (e.g., PIAT)	≥ 0.4
	30+ (e.g., income in 1,000's)	50+ (e.g., PIAT)	≥ 0.1

**Moderate**			
	2-3	50+ (e.g., PIAT)	1.0-2.0
	8-15	50+ (e.g., PIAT)	0.1-0.3
	30+	50+ (e.g., PIAT)	0.02-0.05

**Weak**			
	Anything below moderate that was still significant		

Notes: Ranges are approximate; outliers were included in the category closes to the listed score/range.

## Appendix B

### Statistical Analyses Detailed Methods

First, we conducted simple bivariate associations between each of the 34 predictors and three PIAT outcomes. We then eliminated predictors that did not demonstrate a significant relationship (p < 0.05) with at least one of the three PIAT scores. With the remaining 31 predictors, we created four domain-specific multivariable regression models (i.e. socio-demographic and neighborhood characteristics, family and child care environment, child factors, and health care receipt). When two or more predictors were highly correlated within these models (e.g., "mother smokes daily" and "prenatal cigarette exposure"), we retained the predictor with the strongest bivariate relationship. We also combined family structure with conflict over child rearing into a single variable (partner/high conflict, partner/low conflict, partner/unknown conflict, no partner) because single parents were not asked about conflict over child rearing in the NLSY. After excluding any predictors that were not significantly related (p < 0.05) to at least one of the three PIAT scores, we combined the remaining 22 predictors from all four domain-specific models into one regression model for each PIAT score. We ran the full models and again eliminated any predictors that were not significantly related (p < 0.05) to at least one of the three PIAT scores. Lastly, we re-ran the new full models and excluded any predictors that were not significantly related (p < 0.05) to at least two of the PIAT scores. This final elimination produced three separate parsimonious predictive models, one for each PIAT score. Of note, because no neighborhood or health care receipt predictors were significant in our final models, the remaining predictors were re-categorized into 3 domains (child characteristics, family characteristics, and home environment) for ease of translation into practice; this domain classification is utilized in Tables 2 and 3.

Missing observations from the predictors in our multivariable regression models were handled according to the proportion of missing data [49,50] because this approach (vs. multiple imputations) could be implemented easily in the office setting. For the 26 predictors with ≤ 15% missing data, we recoded missing values as the mode for categorical variables and the mean for continuous variables. For the 7 categorical predictors with > 15% missing data, we assigned a "missing/unknown" category. For the two continuous variables with 16-20% missing data (i.e. income and the HOME emotional subscale), we recoded missing values as the mean to retain the richness of continuous data. Finally, we ran subset analyses on the one predictor [maternal depression: 85% missing] that was missing an overwhelming majority of data to obtain a partial beta co-efficient for this variable.

To gauge the potential effects of missing data on the final model, we compared mean PIAT scores between children with complete data and those with any missing data [49]. Children with missing data were more likely to have lower PIAT scores than those with complete data. However, this difference was not significant after excluding children who had missing data for two predictors that were missing among more than 15 percent of the sample (i.e., income and family structure/conflict). To control for the effects of missing data from these two variables, we included terms for unknowns in the final regression models.

## Pre-publication history

The pre-publication history for this paper can be accessed here:

http://www.biomedcentral.com/1472-6963/11/197/prepub
